# Regulation of Cholesterol Binding to the Receptor Patched1 by its interactions With the Ligand Sonic Hedgehog (Shh)

**DOI:** 10.3389/fmolb.2022.831891

**Published:** 2022-02-02

**Authors:** Changqing Zhong, Beibei Wang

**Affiliations:** ^1^ Centre for Informational Biology, School of Life Science and Technology, University of Electronic Science and Technology of China, Chengdu, China; ^2^ Centre for Advanced Materials Research, Advanced Institute of Natural Sciences, Beijing Normal University at Zhuhai, Zhuhai, China

**Keywords:** Hedgehog signaling pathway, sterol-sensing domain, Martini coarse-grained model, protein-lipid interactions, allosteric communication pathways

## Abstract

The Hedgehog (Hh) signaling pathway is essential in cell development and regeneration, which is activated by the ligand Sonic hedgehog (Shh). The binding of Shh to its receptor Patched1 (PTCH1) releases the inhibitory effect on the downstream protein Smoothened (SMO), a G-protein-coupled-receptor (GPCR) protein. Cholesterol was supposed to function as a secondary messenger between PTCH1 and SMO. However, the molecular mechanism of this regulation process is still unclear. Therefore, microsecond coarse-grained molecular dynamics simulations were performed to investigate the protein-lipid interactions of the PTCH1 monomer and dimer-Shh complex. It was observed that the binding of cholesterols to the monomer is more stable than that to the dimer-Shh complex. It is regulated by the enrichment of Ganglioside lipids around proteins and the conformation of Y446, a residue in the sterol-sensing domain (SSD). The regulation of Shh on the dynamics of PTCH1 was further analyzed to explore the allosteric communication pathways between the Shh and the SSD. Our study provides structural and dynamic details of an additional perspective on the regulation of Hh signaling pathway through the lipid micro-environments of PTCH1.

## Introduction

The Hedgehog (Hh) signaling pathway transmits information between cells, and is critical for embryogenesis and tissue regeneration (Ingham et al., 2011; Briscoe and Therond, 2013). Defects in Hh pathway may lead to birth defects such as holoprosencephaly (Roessler et al., 1996), whereas abnormally activated Hh pathway leads to cancers, most commonly basal cell carcinoma and medulloblastoma (Teglund and Toftgard, 2010). Numerous biophysical and biochemical studies have been reported to explore the molecular mechanism of the Hh pathway (Kong et al., 2019).

The transmitting of Hh signals across the cell membrane is accomplished by two membrane proteins, Patched 1 (PTCH1) and Smoothened (SMO) (Ingham and McMahon, 2001; Haycraft et al., 2005; Wen et al., 2010). PTCH1 works as a receptor, receiving extracellular Hh signals, and then passes to SMO. SMO, a G-protein-coupled-receptor (GPCR), transduces signals across the cell membrane. In the absence of Hh signals, PTCH1 inhibits SMO. The reception of Hh signals, such as the Sonic hedgehog (Shh), activates the Hh signaling pathway by inhibiting PTCH1 and releasing the inhibition of SMO. SMO was not found to have directly interactions with PTCH1 (Taipale et al., 2002), and is activated by cholesterols (CHOLs) (Radhakrishnan et al., 2020), as most GPCRs do (Kiriakidi et al., 2019). Therefore, different mechanisms of the regulation have been proposed on how PTCH1 regulates the activity of SMO, but converged at accessible CHOL working as a second messenger between PTCH1 and SMO (Radhakrishnan et al., 2020).

**FIGURE 1:**
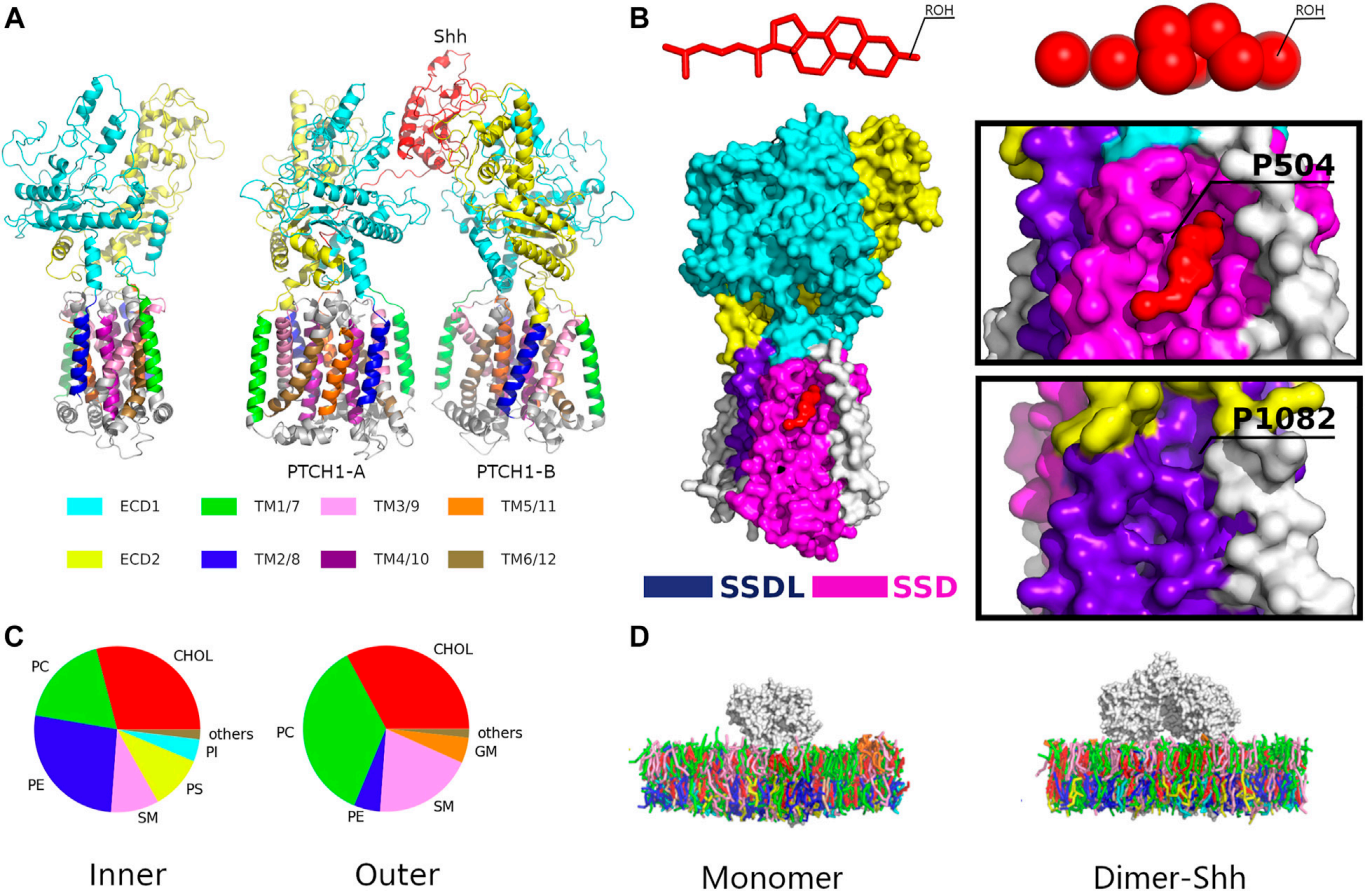
The simulation system. **(A)** Structures of the PTCH1 monomer and dimer-Shh. **(B)** The locations of the SSD and SSDL sites with a close view of Cholesterol binding to the SSD pocket. **(C)** The lipid compositions of the outer and inner leaflets of the membrane model. Lipid types included are phosphatidylcholine (PC), phosphatidylethanolamine (PE), phosphatidylserine (PS), sphingomyelin (SM), phosphatidylinositol (PI), ganglioside (GM), Phosphatidic acid (PA), and cholesterol (CHOL). **(D)** The Martini CG models of PTCH1 monomer and dimer-Shh systems.

The Hh ligand, Shh, is modified by N-terminal palmitoylation and C-terminal cholesterylation (Qi et al., 2018a). Shh binds two PTCH1 receptors (PTCH1-A and PTCH1-B) asymmetrically, forming the PTCH1 dimer-Shh complex. PTCH1 contains two extended ectodomains (ECDs, ECD1 and ECD2) and a transmembrane domain (TMD) with 12 TM helices (TM 1-12) (Gong et al., 2018; Qi et al., 2019) (Figure 1A). TMs 1-6 and TMs 7-12 form two pseudo-symmetrical segments. A sterol-sensing domain (SSD), conserved in several cholesterol-related proteins (Davies and Ioannou, 2000), was identified at TMs 2-6, and an SSD-like (SSDL) domain at TMs 8-12 (Figure 1B; Supplementary Figure S1). CHOLs were found to interact at multiple sites of both SSD and SSDL (Qi et al., 2019) with different binding affinities (Ansell et al., 2021). Two ECDs are located between TMs 1 and 2, and TMs 7 and 8 respectively, and involved in ligand binding. Densities of sterol-like molecules in the ECD suggest a hydrophobic conduit (Zhang et al., 2018). The structures of the 2:1 PTCH1 and Shh complex (termed dimer-Shh here) (Qi et al., 2018a; Qian et al., 2019; Rudolf et al., 2019) show that Shh binds ECDs of two PTCH1s with its C-terminal CHOL bound in the ECD1, and the N-terminal palmitate and 15 residues inserted into the ECD cavity of PTCH1-A (Figure 1A).

PTCH1 has homology with Niemann–Pick C1 (NPC1), a lysosomal membrane protein that transports CHOL from the lumen of the lysosome to the cytoplasm, and resistance-nodulation-division (RND)-family transporters, which efflux toxic molecules out of gram-negative bacteria (Gong et al., 2016; Pfeffer, 2019). Supported by the structural evidence (Qi et al., 2018b; Zhang et al., 2018), PTCH1 was supposed to be a potential CHOL transporter that transfers CHOLs from the cell membrane to the outside of the cell, to reduce accessible CHOLs (Zhang et al., 2018; Kinnebrew et al., 2019). The insertion of the N-terminal palmitate of Shh would block the hydrophobic tunnel, thus inactivating PTCH1. However, Shh, lacking of the N-terminal palmitate and C-terminal CHOL, can also inhibit PTCH1, although the potency is significantly reduced (Pepinsky et al., 1998; Chen et al., 2004). It indicates that the PTCH1-Shh interactions play a role in the regulation of Hh signaling pathway.

In this study, therefore, we resorted to molecular dynamics (MD) simulations to elucidate how the protein-protein interactions of PTCH1 and Shh regulate the accessible CHOL. MD simulations with the Martini Coarse-Grained (CG) force field (Marrink et al., 2007) have been widely used to get insight of the structural details of protein-lipid interactions (Corradi et al., 2018; Corradi et al., 2019; Sejdiu and Tieleman, 2020). Microsecond (μs) CG MD simulations were performed for the PTCH1 monomer and dimer-Shh systems inserted in asymmetric plasma membranes. Differences in lipid micro-environments and CHOL binding between the two systems may illustrate how the binding of Shh regulates accessible CHOLs.

## Methods

**System setup.** The cryo-EM structure of human PTCH1 (PDB ID: 6DMB) (Gong et al., 2018) and human PTCH1-Shh-N complex (PDB ID: 6E1H) (Qi et al., 2018a) were used for the initial structures of PTCH1 monomer and dimer-Shh respectively. In order to investigate the binding of CHOLs in the cell membrane to the protein, the bound cholesterols were removed. The N-terminal palmitate moiety of Shh was not included in the simulation system too, to study the function of the PTCH1-Shh interactions.

The autopsf tool of VMD (Humphrey et al., 1996) was used to repair the missing side chains. Then the atomistic models were converted to Martini CG models using martinize.py, and then CG models were inserted into the CG membrane by insane.py (Wassenaar et al., 2015) with the lipid composition according to the plasma membrane (Ingolfsson et al., 2014) (Figures 1C,D; Supplementary Figure S2; Supplementary Table S1). Finally, the systems were solvated with Martini water models, and neutralized and ionized with 0.15 m/L NaCl. The final simulation systems consist of approximate 55,000 Martini beads.

**Simulation details.** All simulations were carried out with GROMACS version 2019.2 (Van Der Spoel et al., 2005) and Martini 2.2 force field (Marrink et al., 2007). The systems were firstly minimized for 2000 steps with steepest descent method, and then equilibrated by 20 ns NVT simulations with incremental time steps of 2 fs, 5 fs, 10 fs, 15 fs, and 20 fs. Production runs were performed with the NPT ensemble of the constant temperature of 300 K by velocity-rescaling thermostat (Bussi et al., 2007) and constant pressure (1atm) *via* Parrinello-Rahman barostat (Berendsen et al., 1984). A cutoff of 12 Å with a shift of 10 Å was used for calculations of van der Waals and electrostatic interactions. Both PTCH1 monomer and dimer-Shh simulations were performed for a total simulation time of 200 μs, 5 replicates of 40 μs, respectively. All the analyses were performed using VMD (Humphrey et al., 1996).

**Protein-lipid interactions.** The protein-lipid interaction was considered when the headgroup of a lipid is within 0.65 nm of any residue of the protein. For CHOL binding to the SSD/SSDL, the cutoff was set to 1 nm instead of 0.65 nm, because CHOL could be mobile even when interacting at specific interaction sites (Rouviere et al., 2017; Hedger et al., 2019; Duncan et al., 2020). All analyses were performed on the last 10 μs of each simulation.

**Allosteric communication pathways.** The *Network View* plugin (Sethi et al., 2009) of VMD were used to identify allosteric communication networks. The dynamic networks were constructed using our MD trajectories, each sampled every 1ns, with residues represented by the backbone beads. Two nodes (excluding neighboring nodes) within a distance of 6 Å for more than 75% of MD trajectories were defined as contact residue pairs and used to calculate the local-contact matrix. The correlation between two nodes was calculated as $C_{ij}=\frac{<\Delta r_{i}\cdot \Delta r_{j}>}{<\Delta r_{i}^{2}{>}^{1/2}<\Delta r_{j}^{2}{>}^{1/2}}\;$ . The dynamic cross-correlation matrices were calculated via MD-TASK python scripts (Brown et al., 2017). The weight of the contact matrix was calculated as $w_{ij}=-\text{log}\left(|C_{ij}|\right)$ .

The length of a path is the sum of the weights along this path, and calculated as $W=-100\sum^{k}\log\left|C_{ij}^{k}\right|$ for a path with *k+1* nodes. The shortest paths were considered as the optimal paths. The optimal paths along the connecting residue pairs between two nodes were obtained by the Floyd-Warshall algorithm (Floyd, 1962). There is often more than one optimal path. The minimum pairwise correlation along a path (Min) may be the rate-determining step.

## Results

In total of 400 μs simulations were produced. Firstly, we analyzed the CHOL binding and interactions in the SSD pocket. Then, the lipid micro-environments were characterized for both systems. Finally, the conformational dynamic couplings were calculated to reveal the regulation of Shh binding on PTCH1.

**FIGURE 2:**
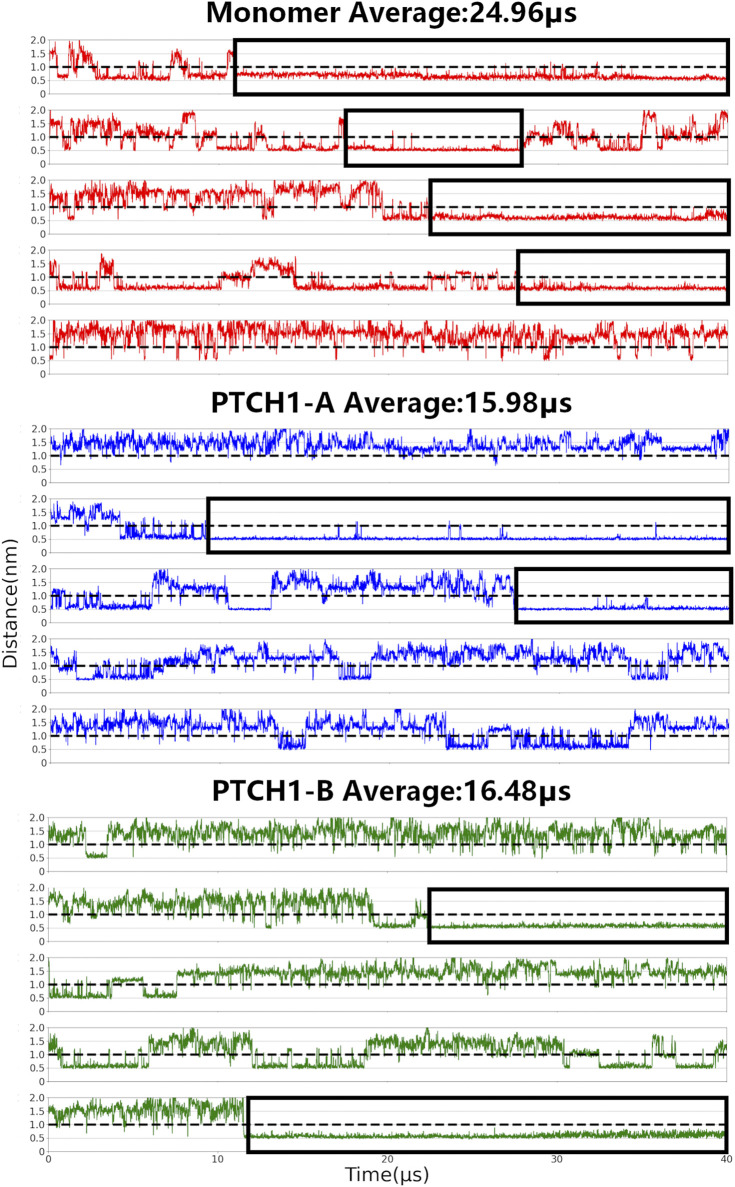
CHOL binding to the SSD. Center-of-mass distances between P504 and its nearest CHOL ROH bead were calculated for all simulations of the PTCH1 monomer and dimer-Shh (PTCH1-A and PTCH1-B) systems.

**CHOL binding to the SSD and SSDL.** As above-mentioned, the SSD of PTCH1 appears a V-shaped cavity, usually occupied by sterol molecules in reported structures (Gong et al., 2018). Comparatively, the SSDL pocket is shallow, and showed low CHOL binding affinity (Supplementary Figure S1). Here, the CHOL binding to the SSD was defined by the distance between the polar hydroxyl group of CHOL (termed ROH in the CG model) and P504, a conserved residue mapping the bottom of the SSD pocket (Boutet et al., 2003; Li et al., 2016; Gong et al., 2018) (Figure 1B). P504 has direct interactions with the ligand and it is involved in all interaction sites of the SSD (Supplementary Figure S1), and the mutant PTCH1-P504L may result in Gorlin syndrome (Boutet et al., 2003). As for the SSDL, the corresponding residue P1082 was used. When the distance is less than 1 nm (the black dotted line in Figure 2; Supplementary Figure S3), it is considered that CHOL binds to the SSD/SSDL. Distinct CHOL affinities of the SSD and SSDL pockets were also observed in our simulations (Figure 2; Supplementary Figure S3). CHOL binding to the SSDL is remarkably weaker and shorter than that to the SSD. There is a CHOL binding to the SSD in about 60%/40% of the simulation time for the monomer and dimer-Shh systems respectively. While, in case of SSDL, the percentage is 11%/18%.

Our simulations also show that CHOL binding to the SSD is also different between the monomer and dimer-Shh simulation systems. For the monomer system, CHOLs bind to the SSD for an average of 25 μs in the 40 μs simulations, and in 4 out of 5 simulations, the binding time of one CHOL lasts more than 10 μs. In simulations of the dimer-Shh system, comparatively, the binding time of CHOLs to the SSD is about 16 μs on average, with stable interactions lasting for more than 10 μs appearing in 2 out of 5 simulations. It is worth to note that CHOL binding affinity to SSD in the PTCH1-A and PTCH1-B is very similar. The populations of the number of residues, that have interactions with CHOLs, also show that in the monomer system, CHOLs have more interactions with the protein than that in the dimer-Shh system (Supplementary Figure S4). In summary, our simulations illustrate that the PTCH1 monomer presents a stronger CHOL binding to SSD than the dimer-Shh.

**FIGURE 3:**
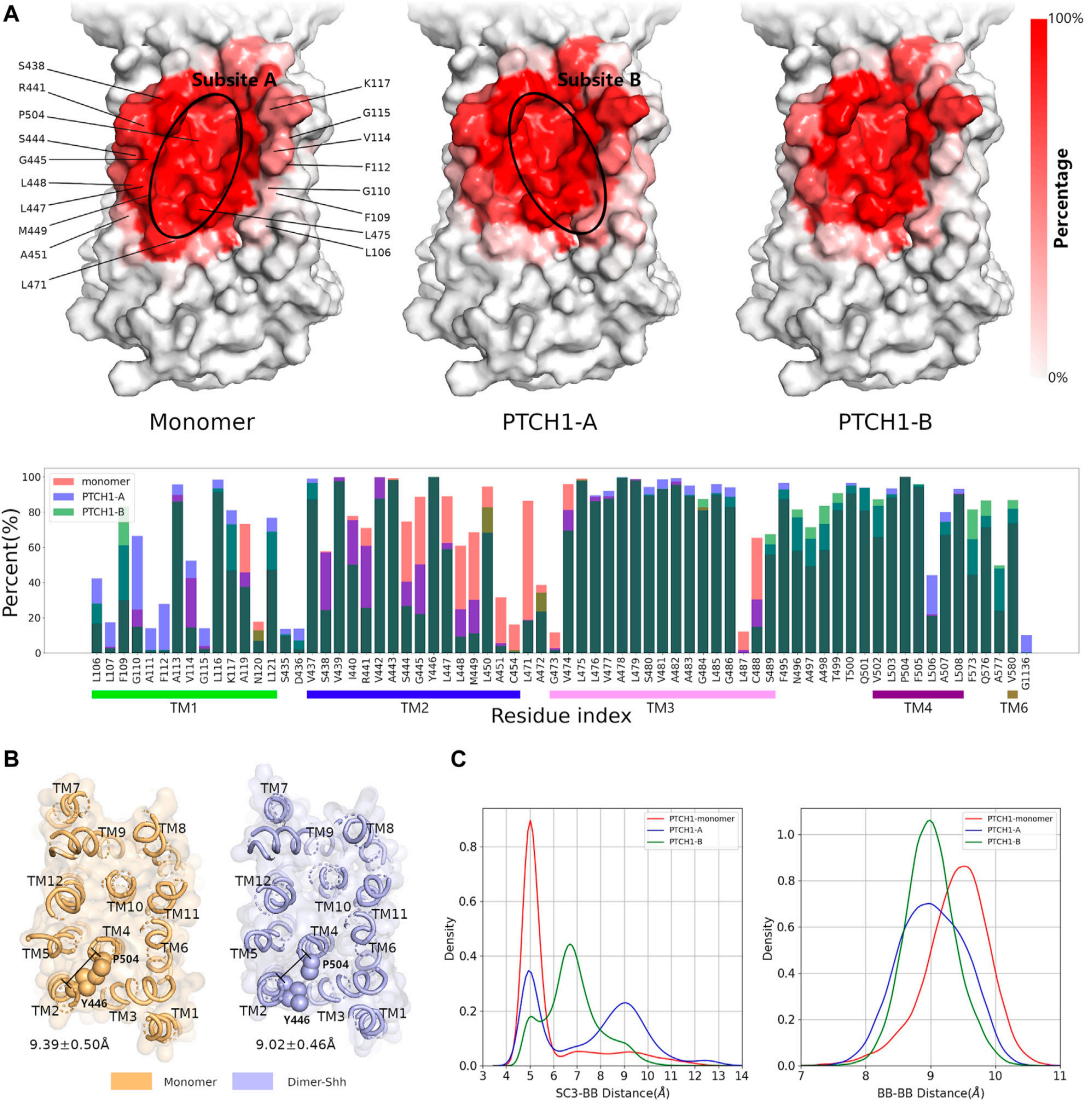
Interactions between CHOL and the SSD. **(A)** Frequencies of residues in the SSD involved in interacting CHOLs for the monomer and dimer-Shh (PTCH1-A and PTCH1-B) systems. The darker the red, the higher the frequency. **(B)** The conformation of Y446 in monomer and dimer-Shh systems. **(C)** Distributions of distances between sidechains (SC3 bead) of Y446 and the backbone (BB) of P504, and backbones of Y446 and P504.

**Interactions between the CHOL and SSD pocket.** The frequencies of interactions between CHOLs and residues involved in the SSD were further calculated, to illustrate the participations of different residues (Figure 3A). For both the monomer and dimer-Shh systems, the residues of TM3 (residues 465-490) and TM4 (residues 495-520), making up the bottom of the SSD cavity, contribute the most in interacting with the polar head of CHOL. The difference presents the contributions of residues on the edge of the SSD, and the hydrophobic tail of CHOL shows distinct binding preferences, termed subsites A and B (Figures 3A; Supplementary Figure S5) in simulations of the monomer and dimer-Shh systems. The subsite A locates at the left edge of the SSD in Figure 3A, involving the residues of TM2 (residues 430–455), while the subsite B locates at the right edge of the SSD in Figure 3A, involving the residues of TM1 (residues 100–125). In simulations of the monomer system, CHOLs prefer the subsite A. In contrast, in the dimer-Shh simulations, CHOL binding to the subsite B is favored.

The conformational changes of the SSD pocket in the monomer and dimer-Shh simulations were further compared. The critical conformational change of Y446 was identified to play a key role in CHOL binding. Mutations near Y446, such as inserting an alanine between Y446 and L447, cause serious diseases (Gu et al., 2006). In monomer simulations, the distances between the backbones of Y446 and P504 are slightly larger than those in dimer-Shh simulations, with the peak of the distance population moving from about 9.5 Å to 9.0 Å (Figures 3B,C). On the contrary, the distribution of distances between the sidechains of Y446 and P504 presents a sharp peak at about 5.0 Å in monomer simulations, while in dimer-Shh simulations, the distribution is wide and ranges from 4.0 Å to 13.0 Å (Figures 3B,C). In monomer simulations, the conformation of Y446 sidechain may benefit the *π*-*π* interactions between Y446 and CHOL. However, in dimer-Shh simulations, the Y446 sidechain rotates and points to the center of the SSD pocket, which makes CHOL binding to subsite A unfavorable.

**Lipid micro-environments of the PTCH1 monomer and dimer-Shh systems.** The lipid micro-environments are fingerprints of membrane proteins (Corradi et al., 2018). The depletion enrichment (D-E) index was defined to describe the tendency of lipids to be enriched (>1) or depleted (<1) around membrane proteins (Corradi et al., 2018; Sejdiu and Tieleman, 2020). The number of lipids in contact with proteins converges after about 30 µs (Supplementary Figure S6), so the last 10 μs trajectories were used to calculate the D-E indices (Table 1; Supplementary Tables S2,S3).

**TABLE 1:** The D−E index of the first lipid shell, which is within 0.7 nm of proteins.

	PC	PE	CHOL	SM
PTCH1 monomer	0.48 ± 0.09	1.47 ± 0.10	1.01 ± 0.07	0.18 ± 0.07
PTCH1 dimer-Shh	0.58 ± 0.04	1.40 ± 0.09	1.02 ± 0.07	0.25 ± 0.09

—	**GM**	**PS**	**PI**	**PA**
PTCH1 monomer	3.54 ± 0.64	1.60 ± 0.39	3.38 ± 0.47	2.40 ± 1.75
PTCH1 dimer-Shh	3.10 ± 0.64	1.70 ± 0.30	3.30 ± 0.66	2.55 ± 0.83

Within the first lipid shell, we observed the depletion of PC and SM, and the enrichment of PE, GM, PI, PS, and PA in both systems. Highly unsaturated lipids are enriched around the protein, correspondingly, lipids with low unsaturation are depleted. In spite of the overall consistency, there are notable differences of the D-E indices of SM and GM. The D-E index of SM in the dimer-Shh system is about 30% larger than that in monomer system, while the enrichment of GM in the dimer-Shh system is lower than that in the monomer system.

**FIGURE 4:**
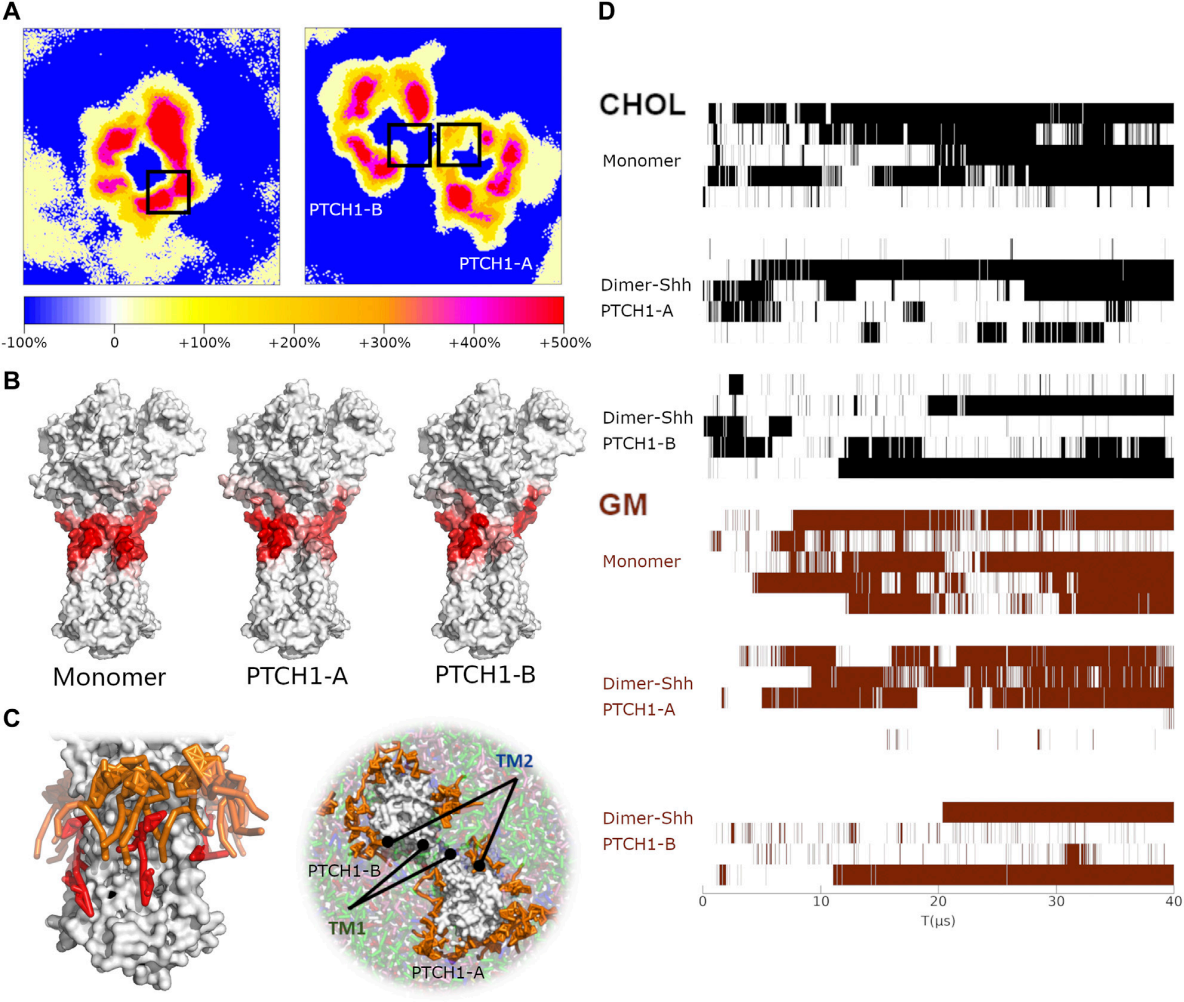
The correlation of GM clusters and CHOL binding. **(A)** 2D density maps of GM for the PTCH1 monomer and dimer-Shh systems, calculated by averaging the last 10μs simulations. The black boxes identify the location of SSDs. **(B)** The hotspots maps of the GM-PTCH1 interactions in the monomer and dimer-Shh (PTCH1-A and PTCH1-B) systems. **(C)** Snapshots of the monomer and dimer-Shh systems. GMs are colored in orange, and CHOL in red. **(D)** Variations of the CHOL binding to the SSD and the GM interacting with the SSD over the simulation time. For the CHOL binding, when the distance of P504-ROH is less than 1 nm, it is marked in black. For the GM interactions, when the residues of TM1 and TM2 have interactions simultaneously with GMs (minimum distance less than 0.65 nm), it is marked in brown.

Two-dimensional (2D) density maps of GM show clearly the enrichments of GMs around proteins, and different patterns of GM distributions of the PTCH1 monomer and dimer-Shh systems (Figure 4A). In particular, in the dimer-Shh system, around the SSD appears no enrichment of GMs, which looks more intuitive *via* frequencies of residues involved in interacting the head groups of GMs and the snapshots of the GM-PTCH1 interactions (Figures 4B,C; Supplementary Figure S7). Frequencies are mapped to the surface of the PTCH1 structure, presenting that in the PTCH1 monomer system, GMs have extensive interactions with the residues of SSD, but GMs seldomly distribute around the TM1 in the dimer-Shh system. The enrichment of GMs around the SSD allows the forming of GM clusters, the tails of which interact with the bound CHOL extensively, favoring the stable CHOL binding (Figure 4C).

We also noticed a close relationship between the existence of GMs around the protein and the CHOL binding in our simulations (Figure 4D). The long-term CHOL binding is always accompanied by the strong interactions of GM-PTCH1. It indicates that the lack of GMs around the SSDs in the dimer-Shh system may be one of the factors of the weak CHOL binding to the SSD.

**FIGURE 5:**
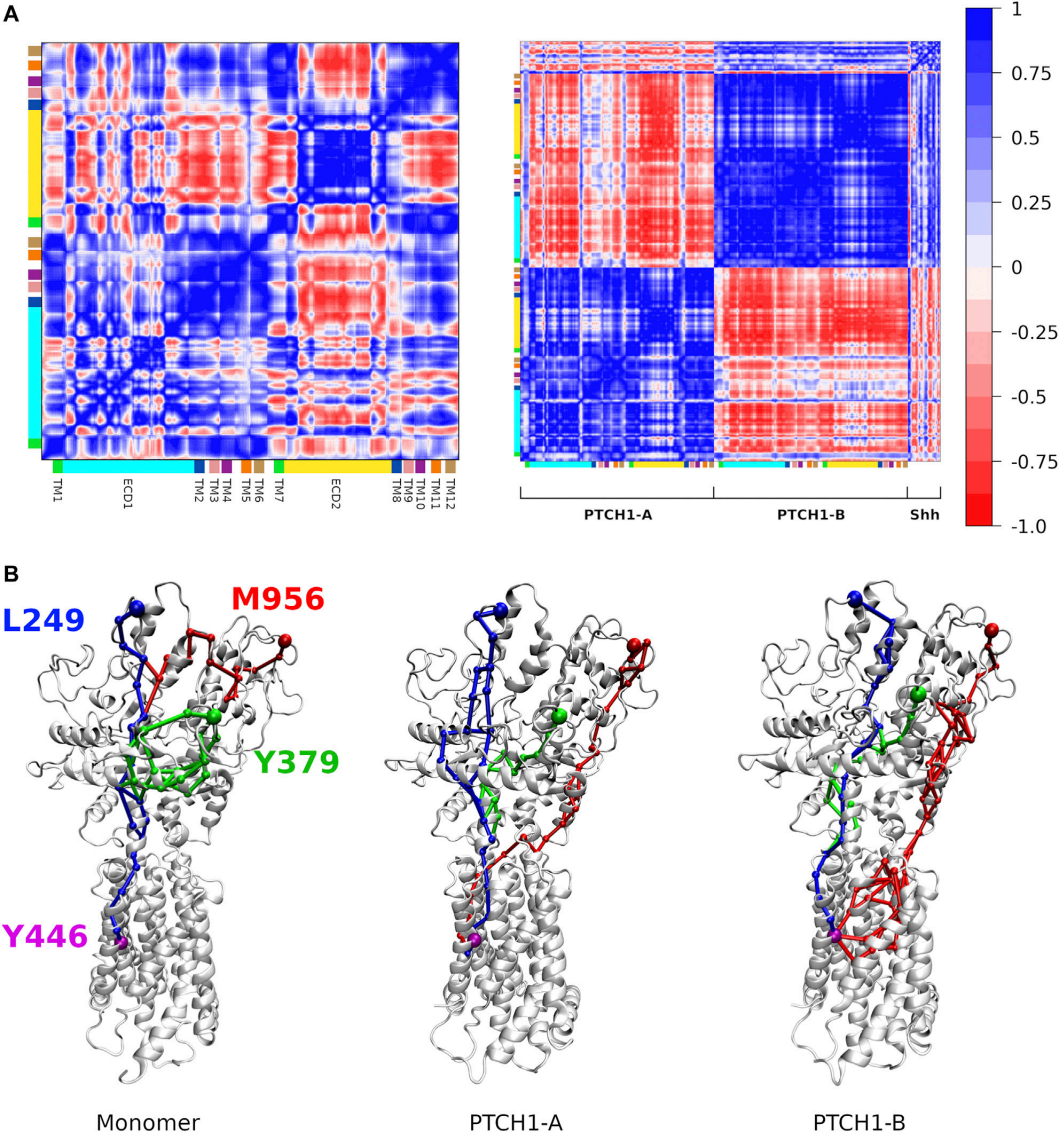
Dynamic communications in the PTCH1. **(A)** Dynamical cross-correlation matrices of backbone beads of the PTCH1 monomer (left) and dimer-Shh (right) systems. **(B)** Allosteric communication pathways between L249/Y379/M956 and Y446, colored in blue, green and red respectively.

**Regulation of the Shh on the conformational dynamics of the PTCH1.** Finally, conformational changes induced by the Shh were investigated. Elastic networks were used for the Martini protein models to maintain secondary structures, so the overall structures of the monomer and dimer-Shh are stable in all simulations, even the relative motion between PTCH1-A and PTCH1-B is limited (Supplementary Figure S8). However, average dynamical cross-correlation matrices (DCCMs) of the PTCH1 monomer and dimer-Shh systems show different patterns (Figure 5A; Supplementary Figure S9). The strong negative coupling of ECD2 and TMD in the PTCH1 monomer is weakened in the PTCH1 dimer-Shh. PTCH1-A is negatively coupled with PTCH1-B in general, but TMs 1-6 of PTCH1-A show weak positive correlation with TMs 1-6 and ECD2 of PTCH1-B. As for the Shh, it is negatively coupled with PTCH1-A, but positively coupled with PTCH1-B in general.

Based on the DCCMs, the allosteric communication pathways between Shh-ECD interface and the SSD were calculated. Three residues at the Shh-ECD interface, L249, Y379, and M956, were selected to represent for the ECD end, while Y446 for the SSD end. Two parameters, the overall weight of the path ( W ) and the minimum pairwise correlation along the path (Min), were used to characterize the optimal pathway (Table 2). A smaller W indicates a shorter path. The L249-Y446 pathways have only limited changes in both systems, they all go directly from the ECD1 to the SSD, using the extended TM2 as a bridge (Figure 5B; Supplementary Figure S10). The Y379-Y446 paths also go through the TM2 bridge, but the path inside ECD1 shifts due to the weak Y379-Y381 dynamic coupling in the dimer-Shh system (Figure 5B; Supplementary Figure S11). In the monomer system, the M956-Y446 pathways also involve the ECD1 and the TM2 bridge. However, in the dimer-Shh system, the M956-Y446 pathways go from the ECD2 directly to the TMD by the T143/Y142-Y971 bridge in most cases, resulting from the weak ECD1-ECD2 correlation (Figure 5B; Supplementary Figure S12). It is worth to note that the optimal paths of M956-Y446 in the dimer-Shh system are much shorter than that in the monomer system (Table 2). To conclude, the binding of Shh and the formation of dimer change the intra-protein dynamic coupling. The detailed results of the correlations and pathways need to be verified by all-atom MD simulations and experiments.

**TABLE 2:** Properties of optimal paths in PTCH1 between the ECD and TMD.

	Monomer		PTCH1-A		PTCH1-B	
	W	Min	W	Min	W	Min
ECD1 (L249)-SSD (Y446)	26	0.96	31	0.95	26	0.91
ECD1(Y379)-SSD (Y446)	19	0.97	50	0.86	24	0.93
ECD2 (M956)-SSD (Y446)	48	0.94	35	0.90	25	0.97

## Discussions

How Shh transmits the signal to SMO through PTCH1 is one of the key questions in the Hh signaling pathway, and is still controversial. In this study, microsecond Martini CG MD simulations were performed for the PTCH1 monomer and dimer-Shh systems, to investigate their characteristic lipid micro-environments. The distinct CHOL binding affinities to SSD and binding sites were observed between two systems. The conformation of a critical residue, Y446, may play an important role in CHOL binding. The lipid micro-environments of the two systems are very similar, but with noticeable difference on the distributions of SM and GM. In the monomer system, GMs are enriched around the SSD, and have extensive interactions with the bound CHOL. However, the enrichment of GMs around the SSD is lack in the dimer-Shh system. Finally, the different patterns of the ECD-TMD coupling were also identified.

As most of GPCRs do, SMO is activated by CHOLs (Kinnebrew et al., 2019; Radhakrishnan et al., 2020). In addition, direct contacts of PTCH1 and SMO are not required for the signaling. Therefore, one of the main proposals is that accessible CHOLs function as a second messenger to pass signals from PTCH1 to SMO (Kinnebrew et al., 2019; Radhakrishnan et al., 2020). It was proposed that PTCH1 regulates accessible CHOLs in two ways: one is to change the distribution/number of accessible CHOLs, the other is to transfer CHOLs out of the cell membrane. Our simulations provide structural details about the regulation of PTCH1 on accessible CHOLs.

Firstly, in the monomer system, CHOL binding to the SSD is more stable and lasts for a longer time than that in the dimer-Shh system. It indicates that the Shh-induced dimerization may release the sequestered CHOLs by PTCH1 monomers, and increase the number of accessible CHOLs in the cell membrane. The stability of CHOL binding to the SSD is related to the population of GMs around the SSD and the conformation of Y446. The tails of GMs interact extensively with CHOLs, which could stabilize the bound CHOL. However, our simulations show clearly that the formation of dimer restricts the approach of GMs to the SSD. On the other hand, the conformation of Y446 in the monomer may form *π*-*π* interactions with the bound CHOL, while its conformational change in the dimer-Shh system blocks the subsite A, and destabilizes the CHOL binding. The conformational change of Y446 may be allosterically regulated by the Shh binding. Secondly, we also noticed that more SMs distribute around the dimer-Shh than the monomer. It is known that SMs sequester CHOLs by forming complexes (McConnell and Radhakrishnan, 2003; Das et al., 2014; Endapally et al., 2019). Depletion of SMs in the cell membrane could also activate SMO (Kinnebrew et al., 2019). Interacting with proteins could also reduce the SM level in the cell membrane, liberating accessible CHOLs from the SM-CHOL complexes. CHOLs in the cell membrane could be divided into three pools: essential, SM-sequestered, and accessible. It is clear that in both the two ways mentioned above, Shh regulates PTCH1 by adjusting the number of accessible CHOLs.

The structural homology of PTCH1 with NPC1 suggests that PTCH1 may work as a transporter, and pass CHOLs out of the cell membrane to an unknown CHOL receptor. However, no CHOL entering the PTCH1 putative hydrophobic tunnel was observed in our simulations. This may be because elastic networks are used for the Martini protein models, and restrict the large-scale conformational changes of proteins (Periole et al., 2009). Further simulations, with smaller force constant of elastic networks or with atomistic force fields, are required to explore PTCH1’s function as a CHOL transporter.

In conclusion, the analysis of protein-lipid interactions, in this study, provides structural details for the improved understanding of how Shh inhibits PTCH1 by regulation of accessible CHOLs, highlights the regulation of the lipid micro-environments on the function of PTCH1, and may open a new avenue for experimental design to reveal the molecular mechanism of the Hh signaling pathway.

## Data Availability

The datasets presented in this study can be found in online repositories. The names of the repository/repositories and accession number(s) can be found below: https://www.jianguoyun.com/p/DSbNbZwQ27-LChjV2Z8E.
