# Supplementary material for: Regulation of Cholesterol Binding to the Receptor Patched1 by its interactions With the Ligand Sonic Hedgehog (Shh)
Source: Front Mol Biosci. 2022 Feb 2;9:831891. doi: 10.3389/fmolb.2022.831891 (PMC8847689; doi:10.3389/fmolb.2022.831891)
Supplement: Supplementary file 1 [file DataSheet2.PDF]

Supporting Information for:

Regulation of cholesterol binding to the receptor Patched1 by its interactions with the ligand Sonic hedgehog (Shh)

Changqing Zhong[a]， and Beibei Wang[b,*]

[a] Centre for Informational Biology, School of Life Science and Technology, University of Electronic Science and Technology of China, 2006 Xiyuan Ave, Chengdu, Sichuan, 611731, P.R China.

[b] Centre for Advanced Materials Research, Advanced Institute of Natural Sciences, Beijing Normal University at Zhuhai, 18 Jinfeng Road, Zhuhai, Guangdong, 519087, P.R. China.

Table S1. The Lipid compositions of the outer and inner leaflets of our membrane model.

Outer leaflet				Inner leaflet			
**Type**		**No.**	**Percentage**	**Type**		**No.**	**Percentage**
CHOL		206	32.85%	CHOL		180	29.22%
DAPC	di-C20:4-C22:5 PC	2	0.32%	DAPC	di-C20:4-C22:5 PC	1	0.16%
DOPC	di-C16:1-C18:1 PC	6	0.96%	DOPC	di-C16:1-C18:1 PC	3	0.49%
PAPC	C16:0/20:4 PC	18	2.87%	PAPC	C16:0/20:4 PC	9	1.46%
PEPC	C16:0/20:2 PC	4	0.64%	PEPC	C16:0/20:2 PC	2	0.32%
PIPC	C16:0/18:2 PC	113	18.02%	PIPC	C16:0/18:2 PC	57	9.25%
POPC	C16:0/18:1 PC	78	12.44%	POPC	C16:0/18:1 PC	39	6.33%
PUPC	C16:0/22:6 PC	4	0.64%	PUPC	C16:0/22:6 PC	2	0.32%
DAPE	di-C20:4-C22:5 PE	5	0.80%	DAPE	di-C20:4-C22:5 PE	23	3.73%
DOPE	di-C16:1-C18:1 PE	2	0.32%	DOPE	di-C16:1-C18:1 PE	13	2.11%
DUPE	di-C20:5-C22:6 PE	1	0.16%	DUPE	di-C20:5-C22:6 PE	6	0.97%
PAPE	C16:0/20:4 PE	8	1.28%	PAPE	C16:0/20:4 PE	37	6.01%
PIPE	C16:0/18:2 PE	5	0.80%	PIPE	C16:0/18:2 PE	27	4.38%
POPE	C16:0/18:1 PE	8	1.28%	POPE	C16:0/18:1 PE	39	6.33%
PQPE	C16:0/20:3 PE	1	0.16%	PQPE	C16:0/20:3 PE	6	0.97%
PUPE	C16:0/22:6 PE	2	0.32%	PUPE	C16:0/22:6 PE	13	2.11%
BNSM	C(d20:1/24:1) SM	12	1.91%	BNSM	C(d20:1/24:1) SM	6	0.97%
DBSM	C(d20:1/20:0) SM	8	1.28%	DBSM	C(d20:1/20:0) SM	4	0.65%
DPSM	C(d18:1/18:0) SM	40	6.38%	DPSM	C(d18:1/18:0) SM	19	3.08%
DXSM	C(d24:1/24:0) SM	16	2.55%	DXSM	C(d24:1/24:0) SM	8	1.30%
PGSM	C(d18:1/22:1) SM	2	0.32%	PGSM	C(d18:1/22:1) SM	1	0.16%
PNSM	C(d18:1/24:1) SM	25	3.99%	PNSM	C(d18:1/24:1) SM	11	1.79%
POSM	C(d18:1/18:1) SM	2	0.32%	POSM	C(d18:1/18:1) SM	1	0.16%
XNSM	C(d24:1/24:1) SM	17	2.71%	XNSM	C(d24:1/24:1) SM	8	1.30%
DPG1	C(d18:1/18:0) GM1	16	2.55%	DAPS	di-C20:4-C22:5 PS	1	0.16%
DPG3	C(d18:1/18:0) GM3	15	2.39%	DUPS	di-C20:5-C22:6 PS	1	0.16%
DPCE	C(d18:1/18:0) CER	2	0.32%	PAPS	C16:0/20:4 PS	31	5.03%
DXCE	C(d24:1/24:0) CER	1	0.16%	PIPS	C16:0/18:2 PS	4	0.65%
PNCE	C(d18:1/24:1) CER	1	0.16%	POPS	C16:0/18:1 PS	14	2.27%
XNCE	C(d24:1/24:1) CER	1	0.16%	PQPS	C16:0/20:3 PS	2	0.32%
PODG	C16:0/18:1 DAG	1	0.16%	PUPS	C16:0/22:6 PS	12	1.95%
PIDG	C16:0/18:2 DAG	1	0.16%	PAPI	C16:0/20:4 PI	7	1.14%
PADG	C16:0/20:4 DAG	4	0.64%	PIPI	C16:0/18:2 PI	8	1.30%
				POPI	C16:0/18:1 PI	9	1.46%
				PUPI	C16:0/22:6 PI	3	0.49%
				POPA	C16:0/18:1 PA	3	0.49%
				PIPA	C16:0/18:2 PA	2	0.32%
				PAPA	C16:0/20:4 PA	2	0.32%
				PUPA	C16:0/22:6 PA	1	0.16%

DPCE	C(d18:1/18:0) CER	1	0.16%
PODG	C16:0/18:1 DAG	1	0.16%
PIDG	C16:0/18:2 DAG	1	0.16%
PADG	C16:0/20:4 DAG	1	0.16%

Table S2. The D-E index of lipids with different heads within 0.7nm, 1.4nm and 2.1nm of proteins.

cut-off(nm)	PC			PE			CHOL			SM		
	0.7	1.4	2.1	0.7	1.4	2.1	0.7	1.4	2.1	0.7	1.4	2.1
Monomer	0.34±0.14	0.85±0.19	0.95±0.16	1.64±0.27	1.22±0.29	0.95±0.16	1.02±0.10	0.88±0.13	0.95±0.11	0.15±0.12	0.69±0.27	0.91±0.24
	0.49±0.12	0.74±0.18	0.88±0.18	1.49±0.25	1.33±0.33	0.88±0.18	0.91±0.10	0.84±0.14	0.96±0.13	0.11±0.11	0.63±0.25	0.87±0.26
	0.45±0.11	0.83±0.16	0.98±0.15	1.35±0.22	1.19±0.32	0.98±0.15	1.12±0.11	0.84±0.12	0.95±0.12	0.17±0.11	0.71±0.25	0.86±0.23
	0.53±0.11	0.81±0.14	0.99±0.15	1.37±0.26	1.21±0.22	0.99±0.15	0.98±0.12	0.84±0.13	0.99±0.11	0.32±0.15	0.61±0.23	1.03±0.26
	0.61±0.13	0.76±0.14	0.85±0.15	1.47±0.28	1.22±0.27	0.85±0.15	0.98±0.11	0.84±0.13	0.93±0.11	0.16±0.13	0.68±0.21	0.87±0.23
Dimer-Shh	0.61±0.09	0.81±0.14	1.09±0.14	1.56±0.15	1.17±0.20	1.09±0.14	0.90±0.07	0.89±0.11	0.93±0.10	0.15±0.09	0.70±0.19	1.02±0.20
	0.62±0.09	0.78±0.13	0.97±0.12	1.41±0.20	1.07±0.19	0.97±0.12	1.07±0.07	0.87±0.09	0.99±0.11	0.25±0.10	0.83±0.18	1.23±0.19
	0.51±0.10	0.98±0.13	1.06±0.13	1.39±0.22	1.13±0.20	1.06±0.13	0.99±0.08	0.88±0.10	0.96±0.10	0.20±0.09	0.69±0.18	0.99±0.20
	0.58±0.09	0.76±0.14	0.97±0.16	1.35±0.17	1.07±0.21	0.97±0.16	1.10±0.07	0.88±0.10	0.95±0.10	0.19±0.08	0.83±0.17	1.08±0.21
	0.57±0.10	0.83±0.13	1.03±0.13	1.27±0.19	1.03±0.21	1.03±0.13	1.01±0.09	0.91±0.10	0.95±0.09	0.42±0.12	0.82±0.16	1.07±0.20

cut-off(nm)	GM			PS			PI			PA		
	0.7	1.4	2.1	0.7	1.4	2.1	0.7	1.4	2.1	0.7	1.4	2.1
Monomer	4.08±0.83	3.62±1.02	1.74±0.66	1.88±0.57	1.37±0.58	0.98±0.39	3.26±1.07	1.04±0.83	0.85±0.72	2.01±2.37	1.38±1.50	1.35±1.48
	4.21±0.68	4.32±0.83	2.18±0.70	2.15±0.55	1.33±0.58	1.06±0.49	2.67±1.17	1.04±1.00	0.92±0.74	1.35±1.70	1.12±1.45	0.92±1.32
	3.87±0.77	4.75±0.96	2.12±0.56	1.02±0.40	1.11±0.59	1.02±0.49	3.44±1.25	1.47±0.91	1.06±0.66	5.87±1.68	0.66±1.31	0.66±1.18
	2.82±0.58	5.74±0.93	1.50±0.57	1.47±0.76	1.05±0.53	0.94±0.45	4.15±1.54	1.06±0.83	0.90±0.65	1.45±1.75	0.72±1.12	0.86±1.41
	2.73±0.57	5.53±0.81	4.46±0.66	1.46±0.48	1.05±0.47	0.91±0.41	3.41±1.16	1.01±0.75	0.67±0.58	1.34±1.56	1.00±1.74	0.85±1.13
Dimer-Shh	4.02±0.47	5.20±0.63	1.16±0.44	1.33±0.37	1.00±0.45	0.95±0.38	3.97±0.74	0.92±0.59	0.58±0.40	1.51±1.45	0.86±1.17	0.93±0.99
	2.57±0.38	5.16±0.53	1.15±0.30	1.76±0.45	1.05±0.35	0.98±0.31	2.51±0.65	0.89±0.63	0.70±0.48	2.56±2.18	0.78±0.95	0.73±0.92
	3.56±0.40	2.80±0.52	1.08±0.41	2.12±0.37	1.18±0.39	1.05±0.37	3.55±0.61	1.39±0.69	0.80±0.51	1.81±1.19	1.27±1.37	0.90±1.05
	2.25±0.29	5.62±0.51	2.11±0.32	1.39±0.30	1.01±0.32	0.93±0.32	3.95±0.72	0.97±0.57	0.73±0.46	3.78±1.15	0.85±1.05	0.61±0.92
	3.10±0.41	4.48±0.61	1.29±0.58	1.89±0.33	0.97±0.34	0.87±0.30	2.51±0.80	1.15±0.72	0.78±0.55	3.08±1.65	1.65±1.37	1.16±1.12

Table S3. The D-E index of lipids with different unsaturation within 0.7nm, 1.4nm and 2.1nm of proteins.

No. unsaturated bonds in two hydrophobic tail (the content)	Distance (nm)	PTCH1-monomer					PTCH1-dimer				
0/0 (8.93%)	0.7	0.59±0.23	0.80±0.19	0.44±0.19	0.60±0.16	0.67±0.21	0.51±0.15	0.35±0.12	0.64±0.11	0.59±0.12	0.69±0.19
	1.4	1.35±0.32	1.35±0.41	1.12±0.37	1.50±0.30	1.37±0.36	1.35±0.23	1.55±0.25	1.00±0.25	1.48±0.20	1.45±0.25
	2.1	1.04±0.31	1.07±0.39	1.30±0.29	0.99±0.28	1.74±0.30	0.93±0.24	1.26±0.29	1.00±0.23	1.24±0.24	1.10±0.28
1/0 (23.33%)	0.7	0.49±0.11	0.43±0.12	0.73±0.13	0.62±0.12	0.29±0.11	0.56±0.08	0.59±0.10	0.59±0.10	0.44±0.08	0.55±0.12
	1.4	0.81±0.18	0.84±0.20	1.06±0.21	0.89±0.21	0.89±0.19	0.94±0.16	1.01±0.13	0.95±0.16	1.02±0.14	0.90±0.16
	2.1	0.97±0.19	0.95±0.18	0.87±0.21	1.07±0.18	0.88±0.17	1.05±0.14	1.05±0.16	0.98±0.16	1.07±0.16	1.00±0.15
1/1 (1.93%)	0.7	0.67±0.75	0.45±0.60	1.04±0.53	0.17±0.38	0.37±0.58	0.44±0.42	0.51±0.49	0.96±0.45	0.47±0.40	0.67±0.47
	1.4	1.15±1.08	0.84±0.74	0.67±0.76	1.15±0.72	0.74±0.82	0.87±0.59	0.49±0.47	0.79±0.62	0.73±0.56	0.96±0.59
	2.1	1.12±0.90	0.90±0.72	0.69±0.59	0.97±0.70	0.87±0.61	1.16±0.59	0.67±0.52	1.12±0.68	1.01±0.48	1.03±0.63
2/0 (17.38%)	0.7	0.60±0.17	0.53±0.19	0.63±0.18	0.64±0.18	0.99±0.20	0.69±0.14	0.79±0.12	0.69±0.17	0.95±0.16	0.71±0.18
	1.4	0.94±0.25	0.81±0.21	0.88±0.24	0.93±0.23	0.90±0.21	0.95±0.17	0.87±0.19	1.05±0.21	0.87±0.24	0.97±0.21
	2.1	1.01±0.22	0.93±0.21	0.99±0.23	0.98±0.21	0.91±0.20	1.11±0.20	0.95±0.18	1.05±0.18	0.95±0.20	1.10±0.20
4/0 (9.01%)	0.7	1.36±0.35	1.66±0.46	1.37±0.35	1.39±0.42	1.26±0.34	2.00±0.30	1.48±0.24	1.41±0.32	1.21±0.28	1.65±0.21
	1.4	1.17±0.42	1.36±0.47	1.25±0.43	1.11±0.40	1.23±0.36	1.11±0.28	0.95±0.30	1.16±0.30	1.04±0.26	0.87±0.28
	2.1	1.15±0.36	1.19±0.39	1.03±0.36	1.03±0.30	1.03±0.33	0.99±0.25	1.00±0.26	1.11±0.26	0.98±0.27	0.97±0.28
4/4 (2.50%)	0.7	3.75±1.48	4.83±1.76	2.49±1.30	3.13±1.27	3.96±1.57	4.40±0.61	3.43±0.73	3.07±0.96	2.91±0.63	2.58±0.88
	1.4	1.50±1.03	2.15±1.03	1.78±1.04	1.74±1.05	2.01±1.14	1.49±0.71	1.09±0.62	1.54±0.72	1.35±0.71	1.51±0.77
	2.1	0.96±0.67	1.43±0.81	1.45±0.90	0.72±0.62	1.10±0.80	0.86±0.45	0.67±0.51	0.86±0.58	0.88±0.47	1.00±0.63
5/0 (2.98%)	0.7	2.77±0.94	1.77±1.08	1.83±0.68	2.67±0.87	1.71±0.70	2.10±0.53	2.52±0.51	2.52±0.66	2.81±0.58	2.24±0.63
	1.4	1.62±0.82	1.15±0.63	1.25±0.76	1.53±0.96	1.18±0.66	1.16±0.51	1.42±0.50	1.53±0.61	1.12±0.53	1.12±0.60
	2.1	1.11±0.69	1.03±0.70	1.21±0.59	0.94±0.61	1.04±0.67	1.14±0.55	0.92±0.43	1.07±0.51	0.84±0.46	1.01±0.47

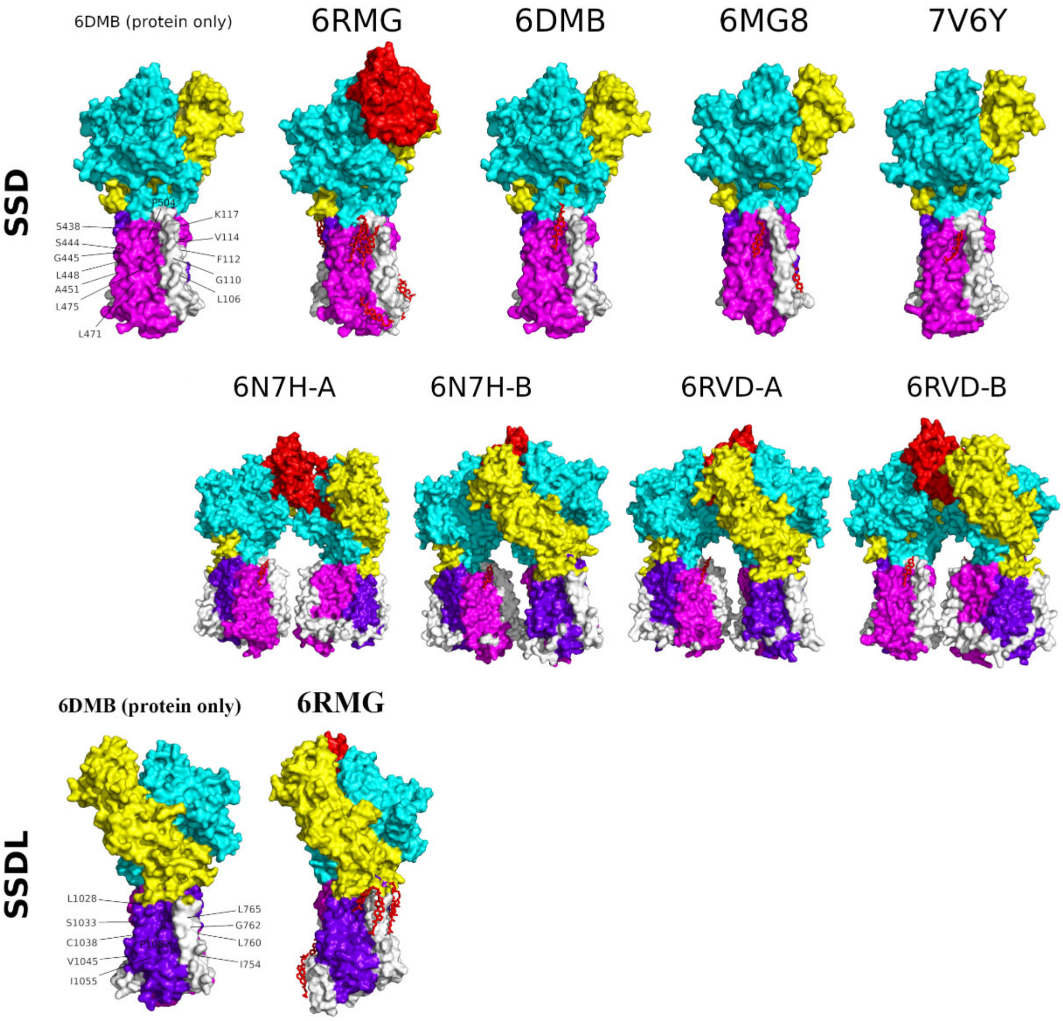

Figure S1. The detailed structures of the SSD and SSDL pockets and their states of CHOL binding in the structures of experimental reports. The coloring strategy is the same as Figure 1, ECD1 in light blue, ECD2 in yellow, Shh in red, SSD in magenta, and SSDL in purple. CHOLs are shown in red sticks. The labels are their PDB ID.

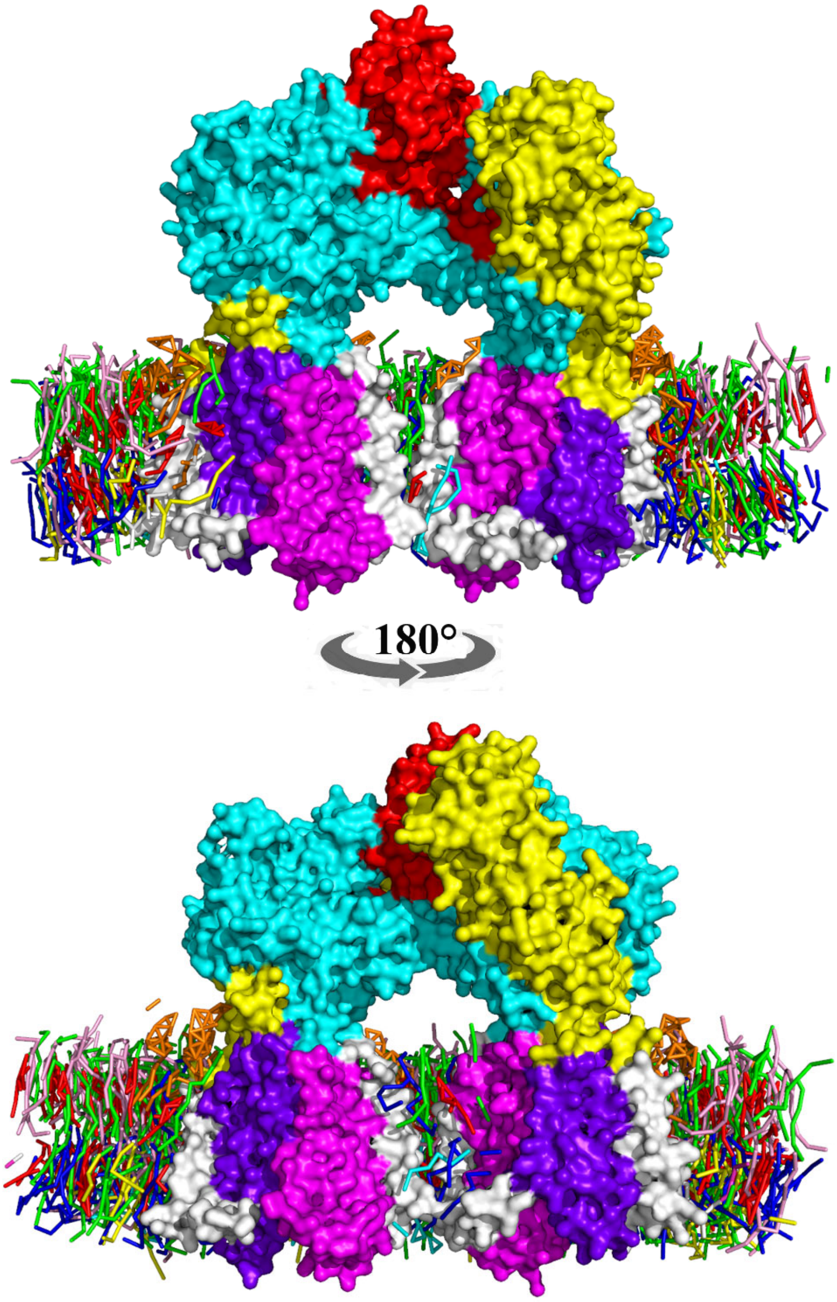

Figure S2. The detailed structure of the dimer in the lipid bilayer. The coloring strategy is the same as Figure S1.

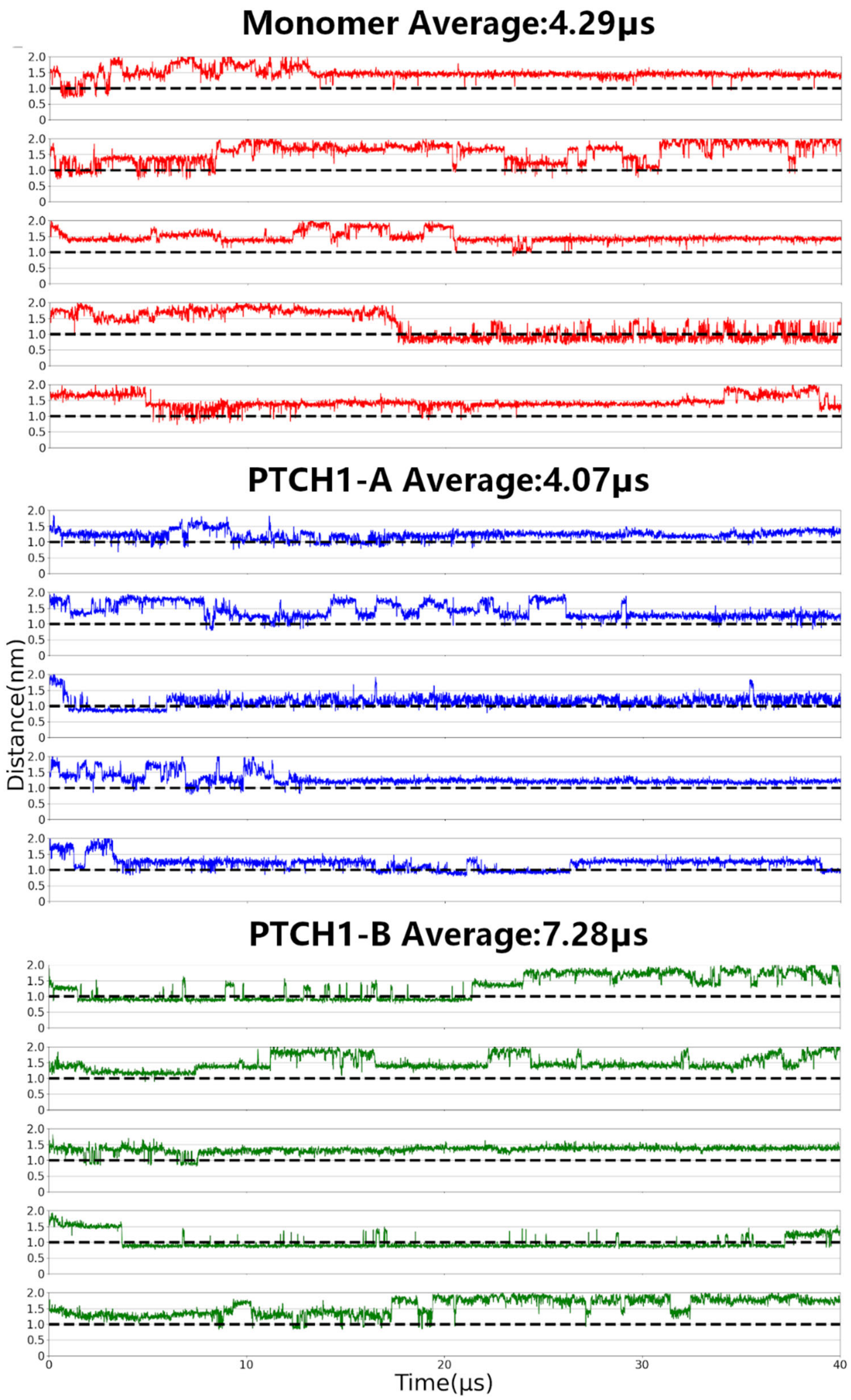

Figure S3. Center-of-mass distances between SSDL P1082 and the nearest CHOL ROH bead in each simulation of PTCH1 monomer, dimer-Shh PTCH1-A and PTCH1-B.

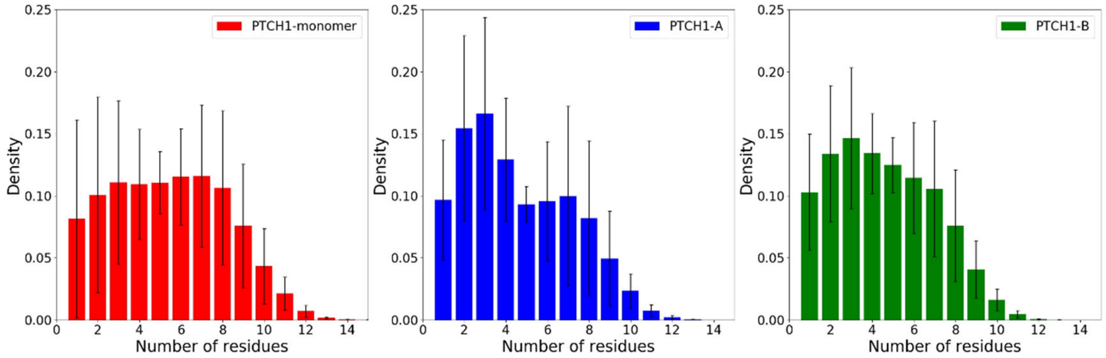

Figure S4. The populations of the number of residues interacting with CHOLs in all simulations of the monomer and dimer-Shh (PTCH1-A and PTCH1-B).

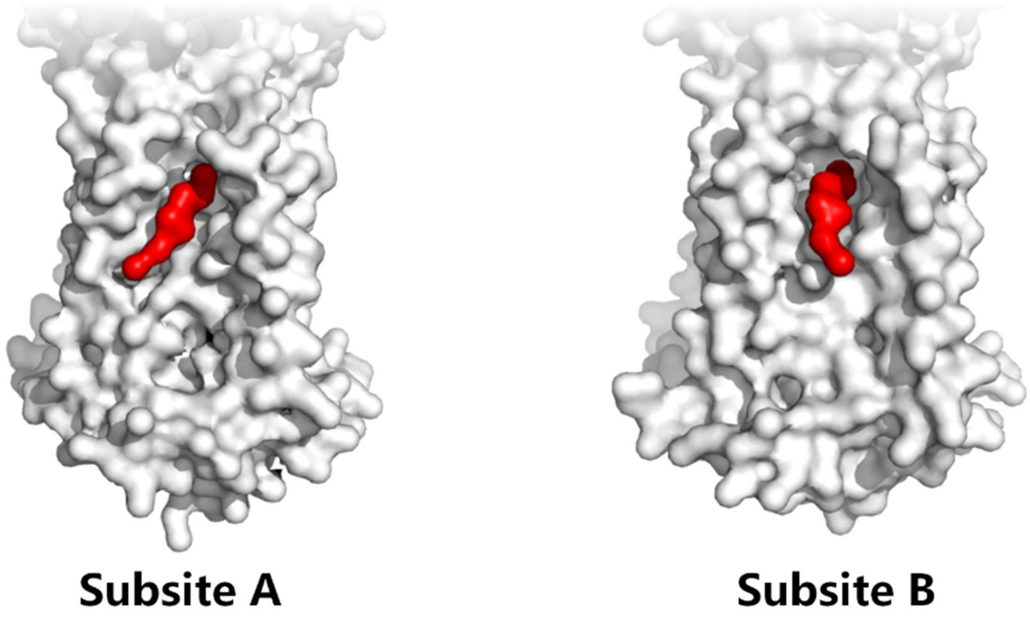

Figure S5. The subsites A and B of CHOL binding.

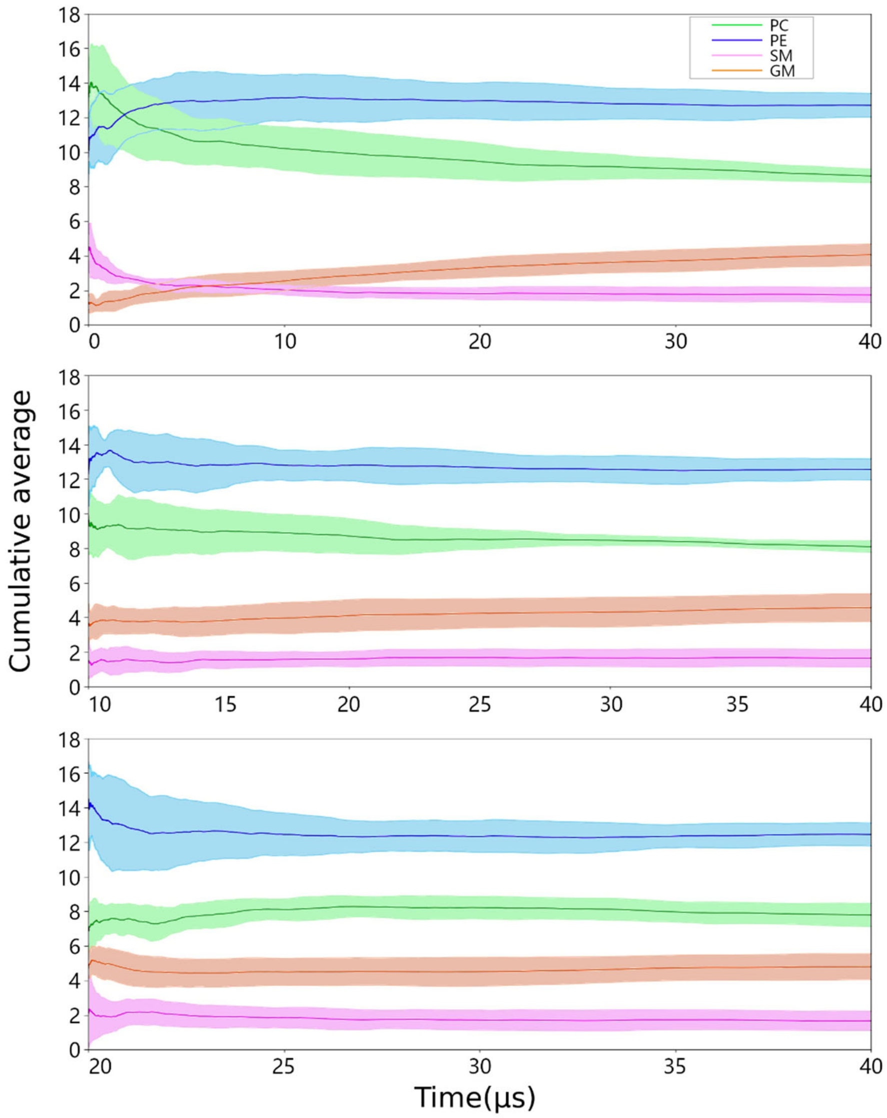

Figure S6. Convergence of the cumulative average of the number of lipids around PTCH1 in the simulations of the monomer (top) and dimer-Shh (PTCH1-A:middle, PTCH1-B: bottom) systems.

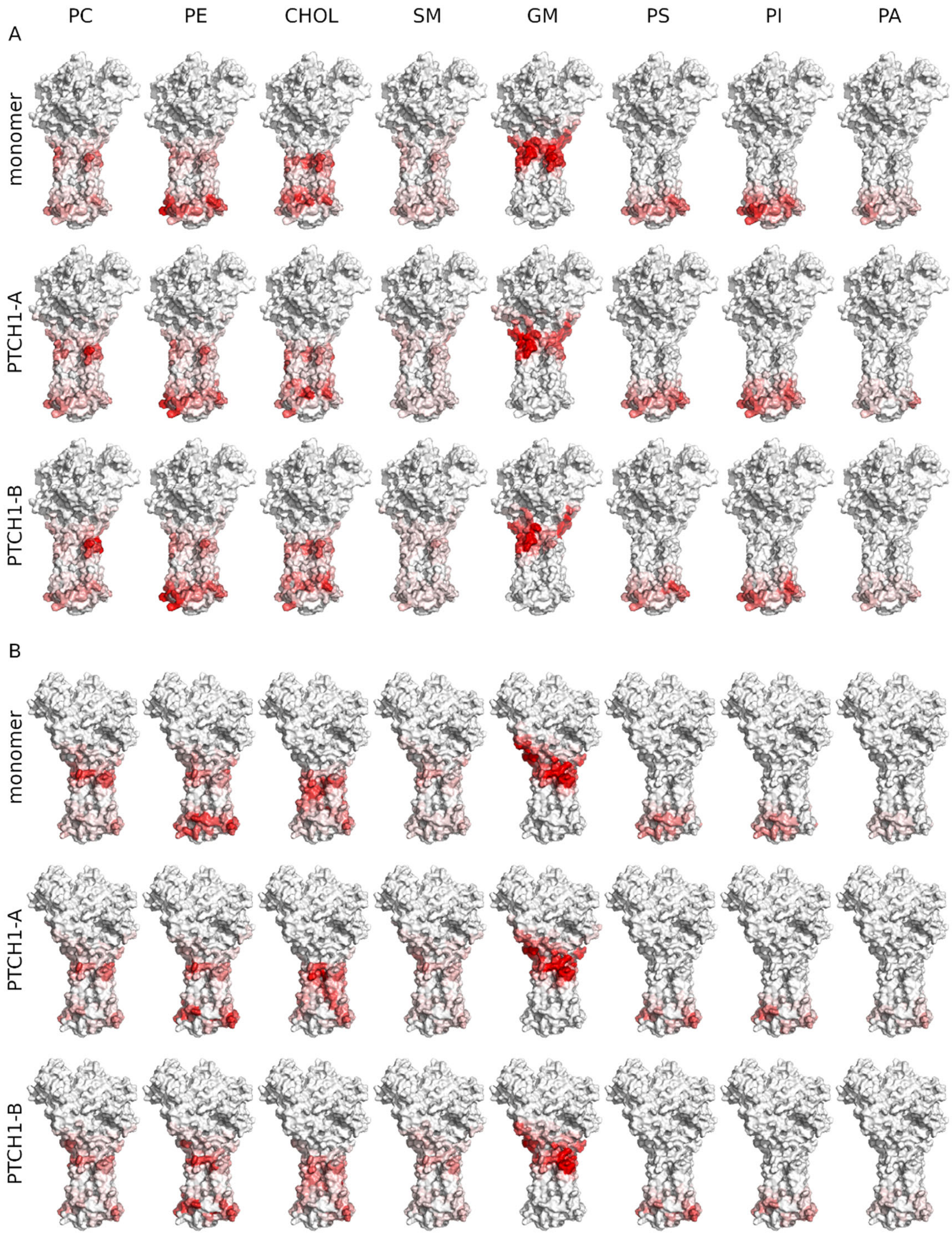

Figure S7. The hotspots maps of residues involved in lipid-PTCH1 interactions, showing the SSD (A) and SSDL sides (B).

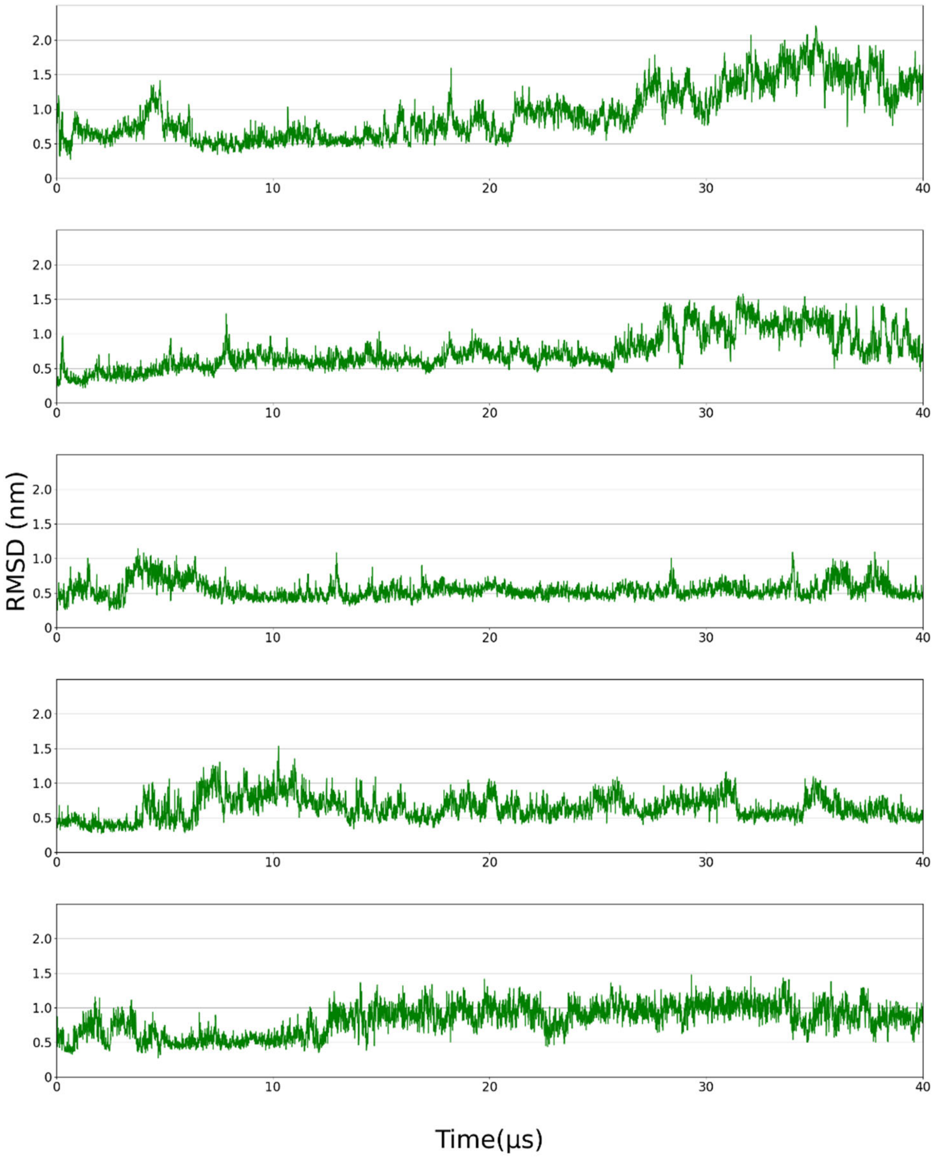

Figure S8. Root mean square deviations (RMSDs) of backbones of PTCH1-B, superimposed on backbones of the initial structure of PTCH1-A.

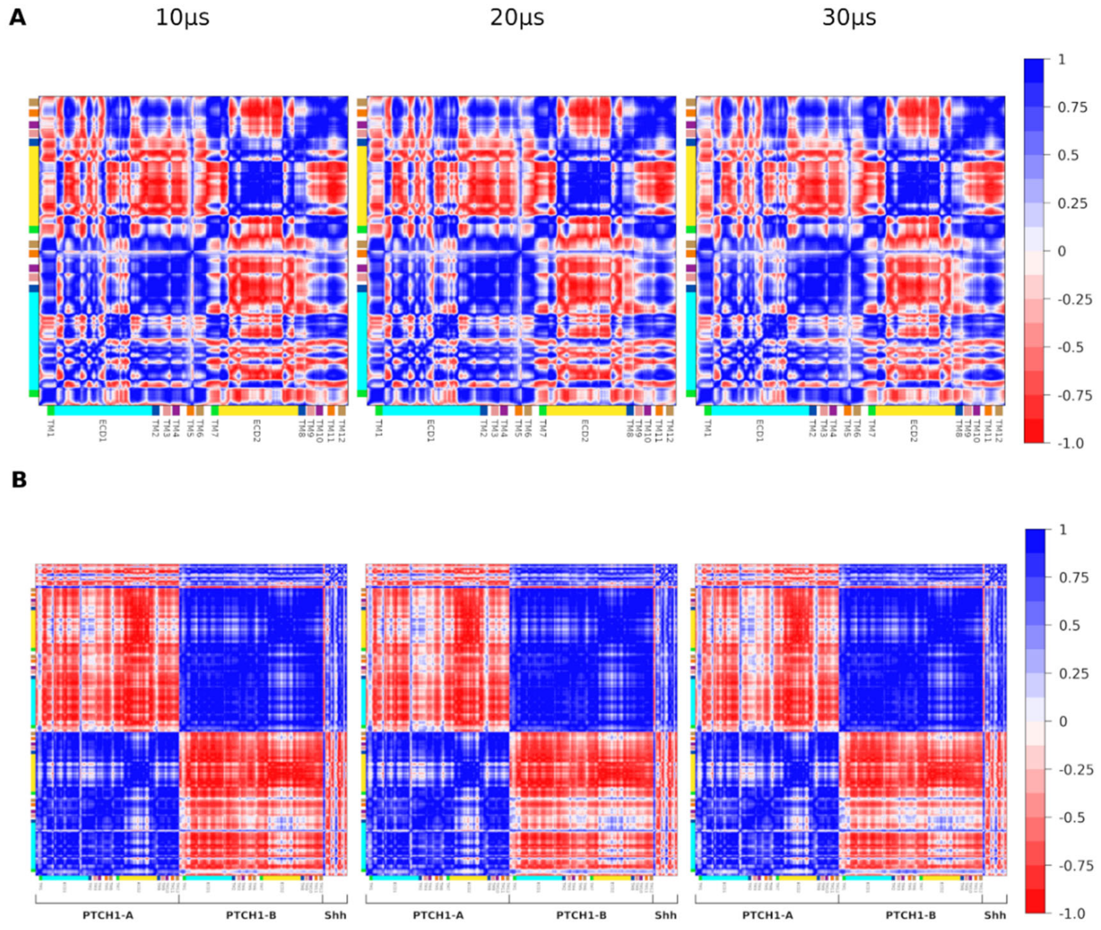

Figure S9. Dynamical cross-correlation matrices for BB beads for (A) PTCH1 monomer and (B) dimer-Shh, using 10, 20 and 30 μs of the simulation trajectories. The DCCMs after 30μs are well converged

Y446-L249

Monomer

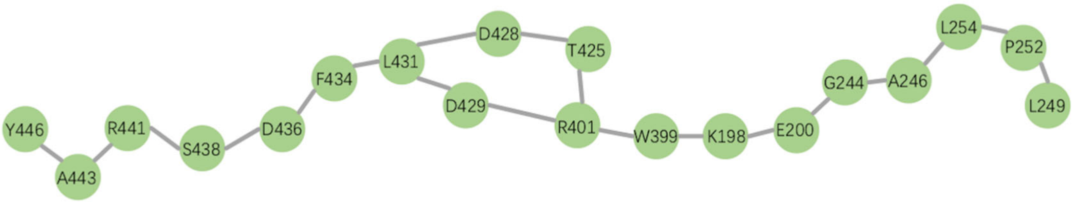

Dimer-Shh
PTCH1-A

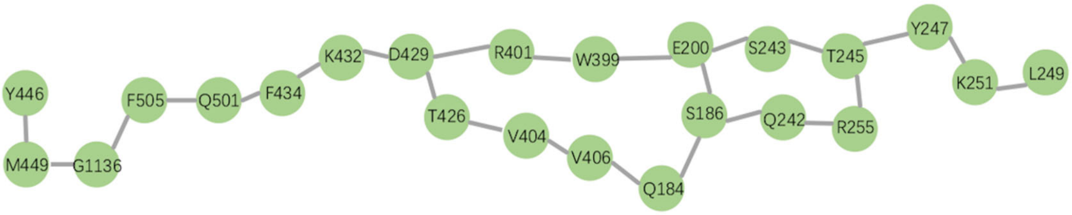

Dimer-Shh
PTCH1-B

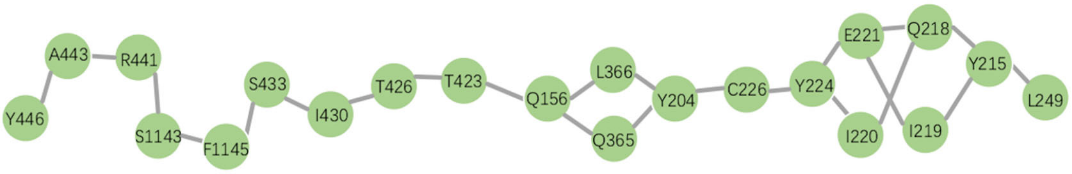

Figure S10. The optimal pathways of Y446-L248.

Y446-Y379

Monomer

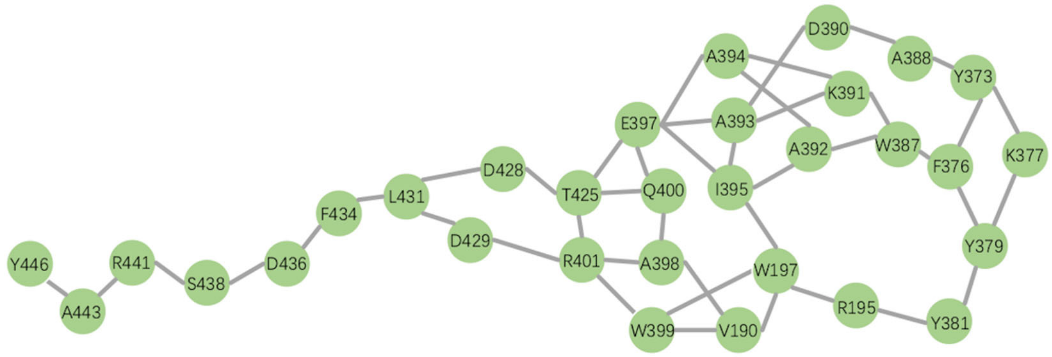

Dimer-Shh
PTCH1-A

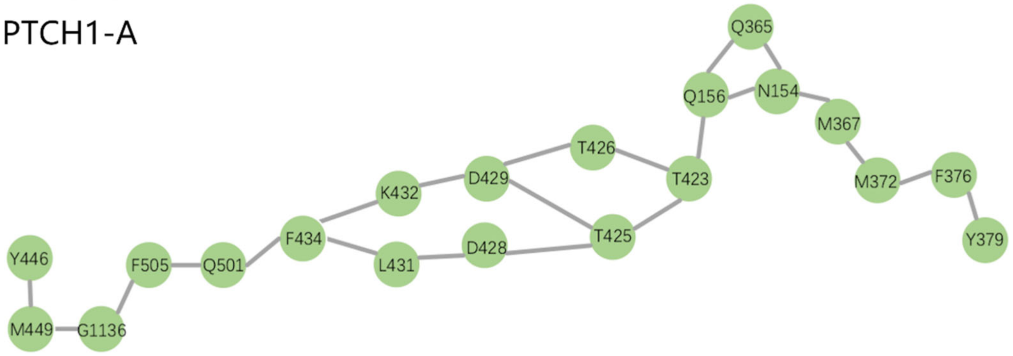

Dimer-Shh
PTCH1-B

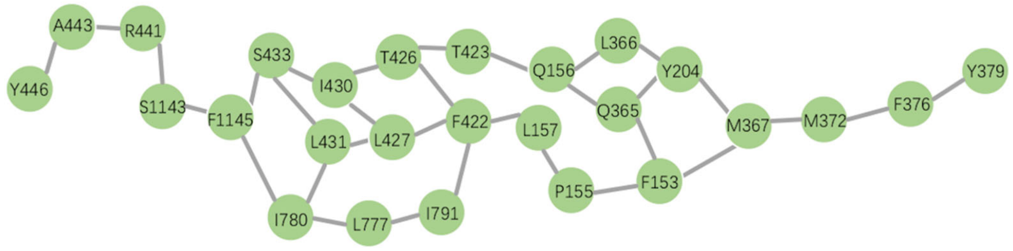

Figure S11. The optimal pathways of Y446-Y379.

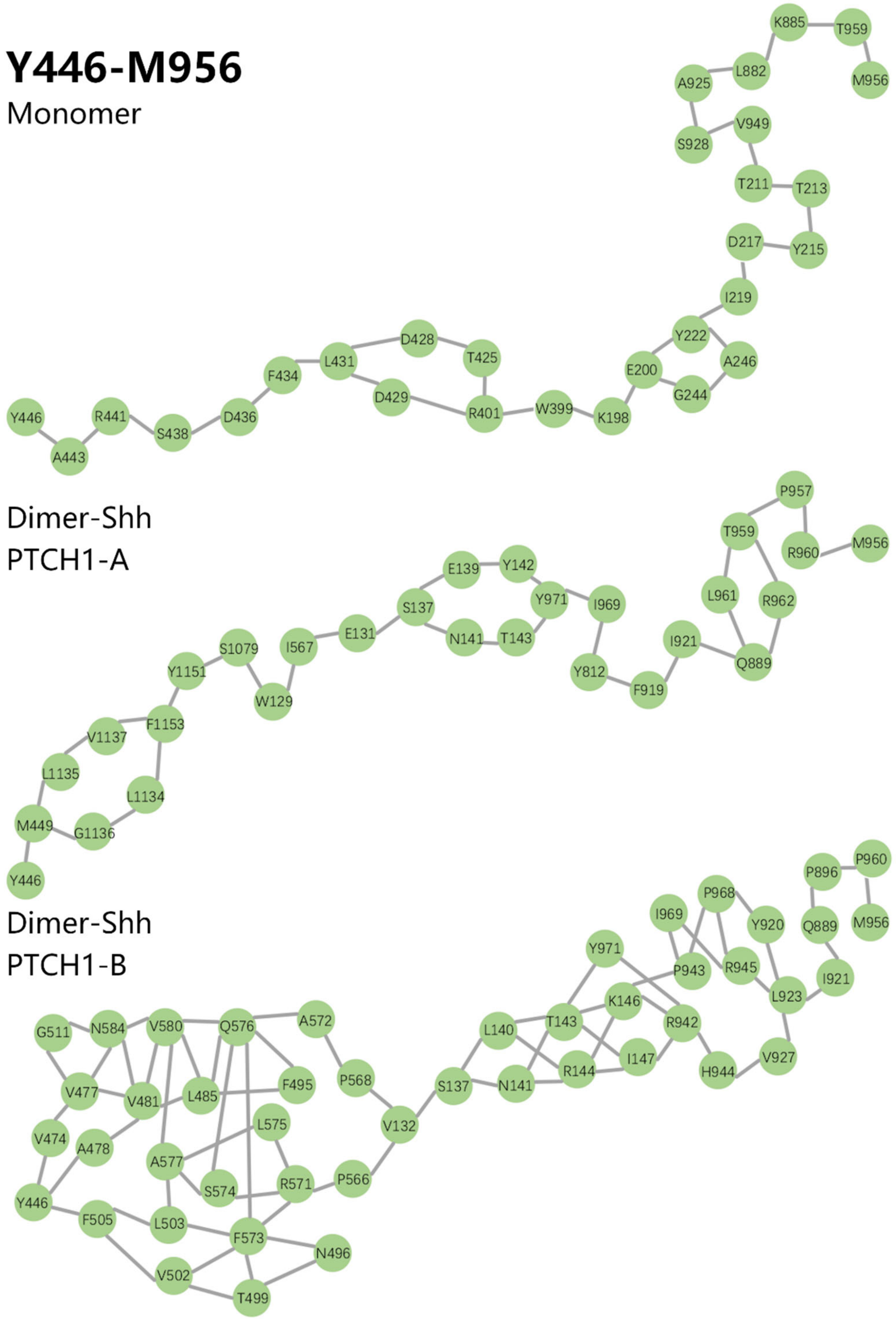

Figure S12. The optimal pathways of Y446-M956.
